# Nomogram for Predicting Semen Parameters Improvement after Microscopic Varicocelectomy in Infertile Men with Abnormal Semen Parameters

**DOI:** 10.3390/jpm13010011

**Published:** 2022-12-21

**Authors:** Xiuping Liu, Dongmei Liu, Chunyu Pan, Hui Su

**Affiliations:** 1 Department of General Surgery, Shengjing Hospital of China Medical University, Shenyang 110004, China; 2 Department of Urology, Shengjing Hospital of China Medical University, Shenyang 110004, China; 3 Department of Sleep Medical Center, Shengjing Hospital of China Medical University, Shenyang 110004, China

**Keywords:** varicocele, varicocelectomy, semen parameters improvement, nomogram, predictor

## Abstract

Objectives: Information on the prediction of improved semen parameters following varicocelectomy is scarce and mostly contradictory. Therefore, we developed and validated a nomogram to predict whether abnormal semen parameters in infertile men could improve following microscopic varicocelectomy (MSV). Methods: From January 2018 to December 2021, 460 consecutive patients who underwent MSV were included. Of them, 336 patients as a development cohort at the Xiang Hua institution. As a validation cohort, Hu Nan Center (124 patients) was used. Clinicopathologic patient information was recorded. The likelihood ratio test using Akaike’s information criteria was employed as the stopping rule, and multivariate logistic regression was utilized to create a prediction model with regression coefficients. The effectiveness of this prediction model was evaluated based on its ability of discrimination, calibration, and clinical utility. Results: The initial total progressively motile sperm count (TPMSC) and vein diameter were predictors of this model. The model demonstrated strong discrimination for the validation cohort, with an area under the receiver operating characteristic (AUROC) of 0.925 (*p* < 0.001), and strong calibration (unreliability test, *p* = 0.522). The decision curve study proved the model’s clinical applicability. Conclusion: According to our research, the improvement of semen parameters in infertile men following MSV was significantly predicted by greater vein diameter and higher initial TPMSC. This nomogram aids in individualized decision-making on the varicocele preoperative treatment plan and may help to enhance the therapeutic result.

## 1. Introduction

Impairment in venous drainage of the testis results in varicocele, which is defined by aberrant dilatation of testicular veins in the pampiniform plexus form [1]. It can eventually have an impact on male fertility and testicular development, as well as cause symptoms of scrotal discomfort and pain, even hypogonadism. Nearly 15% of men with normal sperm quality, 25% of men with impaired sperm quality, and 35 to 40% of men who are infertile suffer from varicocele [2].

The most often used technique is varicocelectomy. Varicocelectomy may enhance semen parameters, spontaneous conception rates, and outcomes of assisted reproductive technologies, according to recent randomized controlled studies and meta-analyses [3,4,5]. Numerous methods have been employed, including open surgery, interventional embolization, microscopic surgery, and laparoscopic surgery [3]. The least invasive procedure should be used in the ideal varicocele treatment plan to achieve high effectiveness and safety.

Due to its benefits of meticulously ligating all veins while sparing the arterial branches, a lower recurrence rate, fewer complications, a higher postoperative semen quality, and a higher postoperative fertility rate, microscopic varicocelectomy (MSV) has gradually come to be the preferred surgical option [6,7,8]. Both the inguinal and subinguinal approaches to MSV are efficient procedures for treating varicocele in infertile men [9]. While varicocelectomy improves semen parameters in only 60–70% of patients and experiences spontaneous pregnancy in only 40–60% of patients, and the fundamental cause of varicocele-related infertility is still unclear [10,11]. The present difficulty is identifying which patients might benefit from surgery the most.

In a retrospective study that included 80 patients who underwent inguinal loupe-assisted varicocelectomy, it was indicated that younger age and higher concentration of preoperative sperm density were prognostic factors for successful varicocelectomy [12]. However, based on a retrospective study that recruited 228 patients who underwent unilateral MSV, Palmisano F et al. [13] found that patients aged older (≥35 years), with higher ultrasound varicocele grading (USVG, grade 3 above), and no concomitant with right subclinical varicocele were more likely to benefit from surgery. Moreover, in a prospective cohort study that included 123 patients treated with subinguinal MSV, Shabana et al. [14] demonstrated that a higher grade of varicocele (II or III), the concentration of sperm density (>8 million/mL), and the ratio of progressive motility (>18%) are significant predictors for excellent outcome after surgery. Data on the prediction of improved semen parameters following varicocelectomy are limited and rather conflicting. As a result, no reliable suggestions can be given. Furthermore, none of studies were validated by external cohorts. By combining and illuminating relevant predictors of critical clinical outcomes and generating a numerical likelihood of the occurrence, a nomogram created from a predictive model is a trustworthy tool for forecasting risk. Therefore, we developed and validated a nomogram to predict whether abnormal semen parameters in infertile men could improve following microscopic varicocelectomy (MSV).

## 2. Methods

### 2.1. Study Design

At two centers of Shengjing Hospital of China Medical University, this retrospective study was carried out (Xiang Hua center and Hu Nan center). The current study included 581 consecutive patients who underwent microscopic varicocelectomy between January 2018 and December 2021. There were 460 patients included in the final study after screening. Hu Nan center (124 patients) served as a validation cohort, while the Xiang Hua center (336 patients) served as a development cohort.

The Ethics Committee of Shengjing Hospital Affiliated China Medical University granted its approval (2022PS719K). ChiCTR2200060504 is the UIN for the clinical research registry. The 1975 Declaration of Helsinki’s ethical principles were followed by the study procedure.

### 2.2. Inclusion and Exclusion Criteria

Inclusion criteria: patients over the age of 18 who underwent MSV (subinguinal or inguinal approach) for infertility with at least one aberrant semen parameter (concentration, total number, motility, or morphology), as well as varicocele that was clinically palpable, are included in the study.

**Figure 1:**
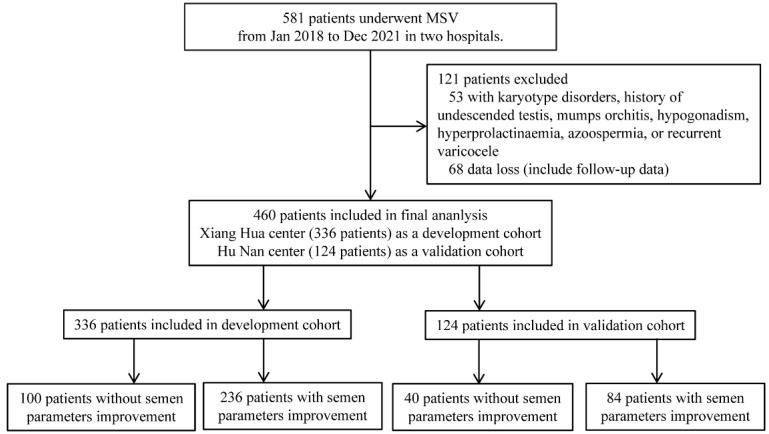
Flowchart of this study.

Exclusion criteria: patients with a history of previous varicocelectomy (recurrent) or other inguinal surgeries (such as hernia repair), chromosomal abnormalities (such as AZF microdeletions or karyotype disorders), reproductive system malformations (such as cryptorchidism), lower urinary tract infections, prostatitis, epididymitis, and seminal vesiculitis, hypopituitarism, hyperthyroidism, Cushing’s syndrome; abnormal levels of luteinizing hormone, follicle-stimulating hormone, or serum testosterone were excluded. The details of flowchart were presented in Figure 1.

The technique of MSV: Testicular artery and lymph vessels sparing and free ligation of gubernacular veins strategy were used.

### 2.3. Measurement of Characteristics and Follow-Up

Patient characteristics (age, body mass index (BMI)), the length of infertility (months), smoking history (never vs. current or former), varicocele data (surgical side (left vs. bilateral), grade (left, II vs. III), diameter of veins (left, mm), testicular volume (left, mL), semen parameters prior to MSV (baseline total progressively motile sperm count (TPMSC, 10^6^/mL), and intraoperative data (surgical approach (subinguinal vs. Inguinal), number of ligated veins).

The physical examination in an upright position led to the diagnosis of varicocele (VC). The VC was categorized based on the Dubin and Amelar grading system: Grade 1: palpable during Valsalva maneuver. Grade 2: palpable at rest. Grade 3: visible and palpable at rest. By using Doppler ultrasonography to quantify all varicoceles, it was possible to establish their existence and determine their true diameters (The maximum venous diameter). The lack of a desired pregnancy after regular, unprotected sexual activity for at least one year is referred to as infertility. A patient is considered to be a current smoker if he has smoked 100 cigarettes during his life. The formula V = L × W × H × 0.71 was used to calculate the volume of the testicles. The total sperm count and the ratio of the progressively motile was used to determine total progressively motile sperm count (TPMSC = total sperm number (10^6^/ejaculate) × progressive motility (PR, %); PR, progressive (a + b motility)] [15]. In post-operative semen analysis, we defined semen parameter improvement as a greater than 50% increase in total motile sperm count (TMSC) within six months of surgery [15]. Semen analysis was conducted repeatedly and assessed in accordance with WHO guidelines [16]. The follow-up assessment was carried out by phone calls or clinical visits.

### 2.4. Statistical Analysis

The data were analyzed using STATA 15.0 (Stata Corporation, College Station, TX, USA), R software (version 3.0.1; https://www.r-project.org/, accessed on 7 November 2022), and IBM SPSS Statistics for Windows, version 22.0 (IBM Corporation, Armonk, NY, USA). The R packages “rms” and “glmnet” were used in this work. With statistical significance being defined as a probability (*p*) value of less than 0.05, all of the statistical significance levels that were reported were two-sided.

The Kolmogorov–Smirnov test was used to establish the normality of continuous variables. The mean and standard deviation were used to describe regularly distributed continuous data, whereas the median (interquartile range) was employed to represent continuous variables that were not normally distributed. The independent-samples *t* test is used to compare the means of two continuous normally distributed variables. The *t*-test for students was used. To compare two continuous non-normally distributed variables, the Mann–Whitney U test was applied. Number was given as the categorical variable (percentage). To compare categorical variables, the Fisher’s exact test and the chi-squared test were used. A predictive nomogram with regression coefficients was constructed using multivariate unconditional logistic regression analysis. With Akaike’s information criteria serving as the stopping rule, the likelihood ratio test was used to apply the backward stepwise selection [17,18].

The effectiveness of the model was assessed in a different validation cohort. The logistic regression model created in the development cohort was utilized to compute the likelihood for each patient in the validation cohort. The area under the receiver operating characteristic (AUROC) curve was evaluated to gauge how well the model discriminated. An AUROC of 0.5 meant there was no prejudice, while an AUROC of 1.0 meant there was total discrimination. Calibration plots, the unreliability test, and the Hosmer-Lemeshow (H-L) chi-square statistic were all used to assess the model’s calibration (*p* > 0.05 indicating good calibration). A slope on the 45-degree line showed that the calibration was perfect. Using decision curve analysis, which evaluated the net benefits at various thresholds, it was possible to assess the clinical relevance of the model.

## 3. Results

There were 336 patients enrolled in the development cohort and 124 individuals included in the validation cohort following screening using the same inclusion and exclusion criteria. Semen parameter improvement after MSV was shown in 70.2% (236/336) of patients in the development cohort and 67.7% (84/124) of patients in the validation cohort, respectively; see more information in Table 1.

**Figure 2:**
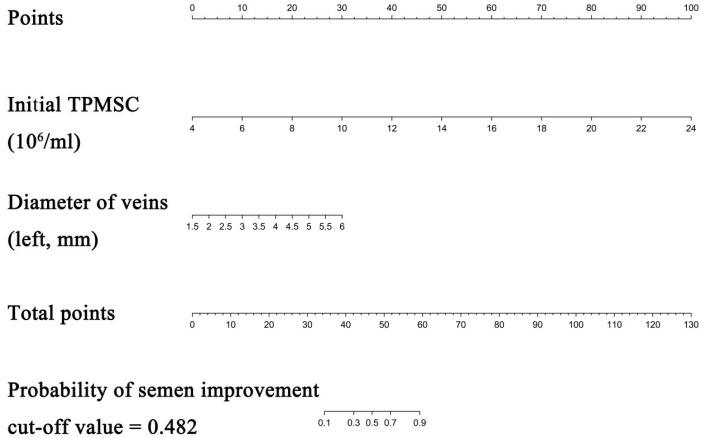
Nomogram to predict semen parameters improvement after MSV. Each clinicopathologic factor corresponds to a specific point by drawing a line straight upward to the Points axis. After summing the points located on the Total points axis, the sum represents the probability of semen parameters improvement after MSV by drawing a line straight down to the risk axis.

Patients with improved semen parameters in the development cohort’s univariate analysis had greater vein diameters and higher baseline TPMSC levels (Table 2). A predictive nomogram with regression coefficients was constructed using multivariate binary logistic regression. The likelihood ratio test using Akaike’s information criteria was employed as the stopping rule. Backward stepwise selection was also used. The final model displays the outcomes (diameter of veins, and baseline TPMSC). Based on these findings, we created a predictive model, from which we constructed a nomogram that predicts an improvement in the semen parameter following MSV (Table 2 and Figure 2).

**Figure 3:**
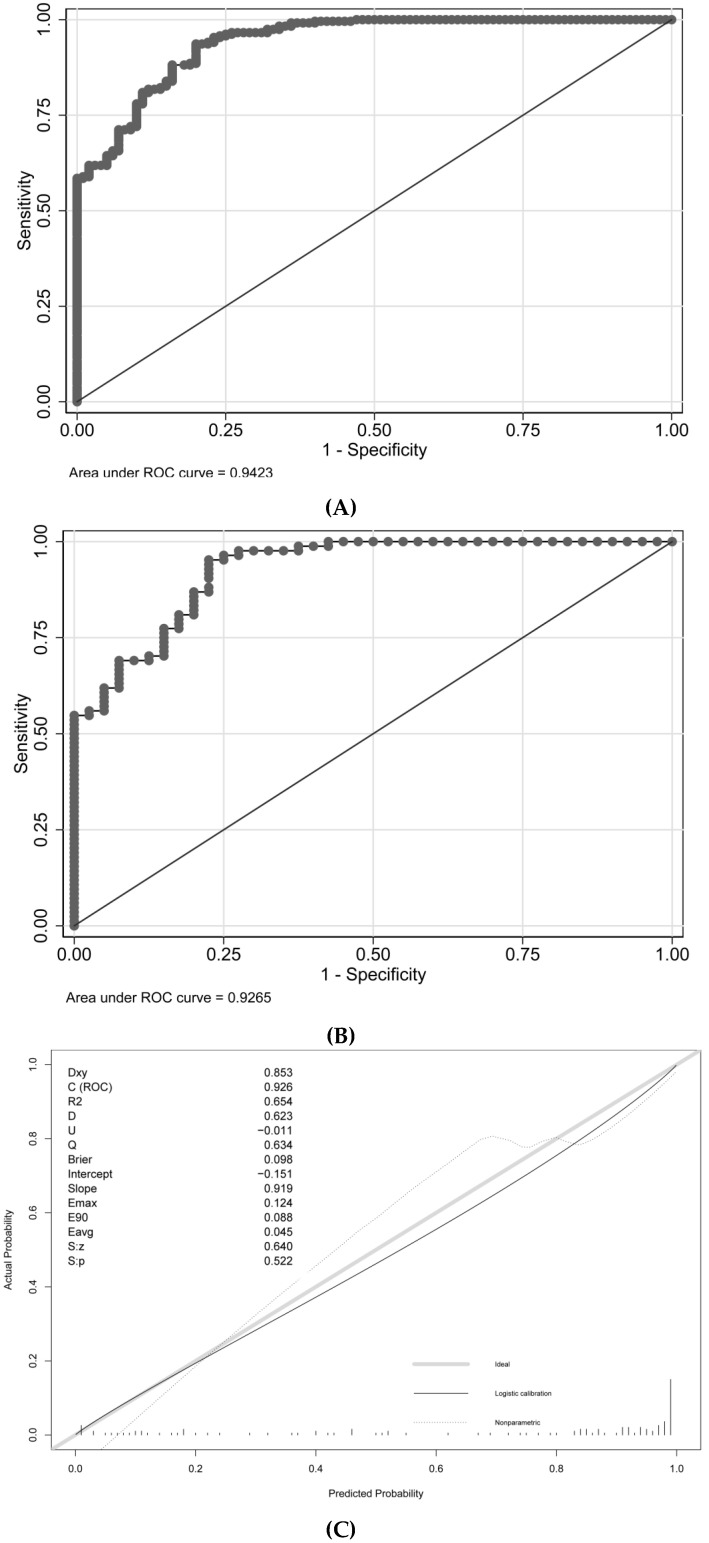

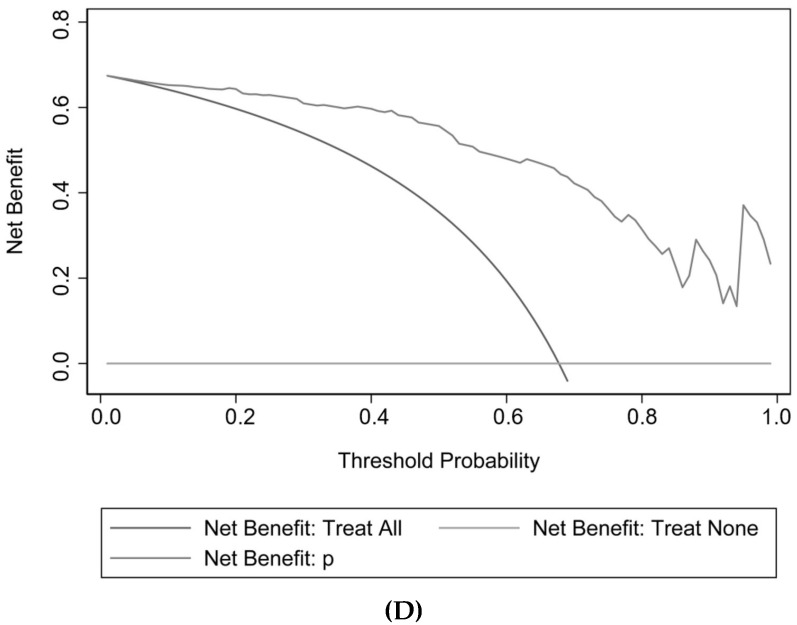
Discrimination, calibration, and decision curve analysis for the model. (**A**) ROC in the development cohort; (**B**) ROC in the validation cohort; (**C**) Calibration plot; (**D**) Decision Curve Analysis.

The cutoff value of probability in this model was 48.2%, with a sensitivity of 93.64% and a specificity of 80.00%. The AUROC values for the development and validation cohorts were 0.9423 and 0.9256, respectively (Table 3 and Figure 3). The unreliability test statistic for calibration in the validation cohort was −0.011, with a *p*-value of 0.522, while the Emax and Eavg values were 0.124 and 0.045, respectively. With a *p*-value of 0.4231 and an H-L chi-square statistic of 10.2, the calibration was deemed to be adequate. The decision curve showed that utilizing this nomogram to predict semen parameter improvement after MSV was more beneficial than using either the treat-all-patients or treat-none schemes if a patient’s threshold probability was between 10% and 100%. The net advantage fell within this range (Figure 3). 

Each clinicopathological characteristic was assigned to a particular point by drawing a line straight upward to the Points axis. The probability of improving the semen parameter was calculated by drawing a straight line from the risk axis straight down to the sum of the points on the Total Points axis. For instance, the baseline TPMSC level is 12 × 10^6^/mL (13 points) and the vein width is 3.5 mm (40 points). The total score for this patient was 53. The estimated chance of semen parameter improvement following MSV was around 72%; the cutoff value was 48.2%. The decision-making process for a treatment plan may benefit from this result. Further information is provided in Supplement Figure S1.

## 4. Discussion

The precise relationship between varicocele and decreased male fertility is unknown. This has been explained by a number of theories, including hypoxia and hemostasis, elevated scrotal temperature, autoimmune, reflux of adrenal metabolites, and enhanced oxidative stress [2]. At least one-third of infertile men do not report an improvement in semen parameters [19,20], it is yet unknown why this is the case and which individuals would benefit from surgery. Therefore, the goal of this study was to develop and validate a nomogram based on a large cohort of infertile men with abnormal semen parameters for predicting semen parameter improvement following MSV. Our research indicates that the improvement of semen parameters in infertile men following MSV is significantly predicted by a greater vein diameter and a higher initial TPMSC.

Total sperm count multiplies the ratio of progressively motile sperm yielded the combined indication known as TPMSC. This study discovered a favorable correlation between greater initial TPMSC and the improvement of semen parameters following MSV. Those who claimed recovery after MSV had a considerably greater TPMSC than patients who did not recover (15.51 million vs. 9.82 million, respectively; *p* < 0.001). Wang et al. [21] found that the semen parameters improvement rate was higher in patients who presented a better baseline TMSC after varicocelectomy; it was highest (increased by 49.68 million) in the TMSC > 10 million group, and lowest in the TMSC 2 million group (increased by 10.20 million). This meta-systemic study, which included nine studies, supports these findings. Therefore, while estimating the semen parameter improvement rate of planned varicocelectomy, the combined indication of TPMSC should be employed.

In this study, improvement in semen parameters following varicocelectomy was significantly predicted by the diameter of veins (as determined by ultrasonography). In agreement with this, Palmisano F et al. [13] discovered that patients with greater USVG (grade 3 and above) were more likely to benefit from surgery based on a retrospective research study that enrolled 228 patients who underwent unilateral MSV. A greater varicocele grade (grade 2 or 3) is a major predictor of excellent results following surgery, according to Shabana et al. [14], who conducted another prospective cohort research with 123 patients who had subinguinal MSV. The physical examination is frequently misleading because to its subjective character and dependence on the surgeon’s expertise, hence in our opinion US grading is superior to palpable examination and more connected to the treatment success. Particularly in patients who are obese, have high-located testes, or have had inguinal surgery in the past may find it to be of limited benefit. However, several investigations have revealed no correlation between varicocele grade and improved semen parameter [12]. The variation in inclusion criteria and the standard of metrics assessed may account for this discrepancy.

The study includes several limitations. First, a single-center retrospective design was used. Second, certain potential predictors were absent despite their lack of widespread acceptance in clinical practice, such as DNA fragmentation index and anti-sperm antibodies. The anti-sperm antibody (ASA) can decrease sperm motility and, therefore, cause male infertility. However, current evidence has shown that positivity for ASA is not a predictor of the outcome after varicocelectomy [22]. Third, a recent study demonstrated that laparoscopic varicocelectomy is also a valid therapeutic approach to improve semen parameters for further assisted reproductive techniques; and it is particularly preferred in those who underwent bilateral varicocelectomy [23]. However, because microscopic surgery is the more preferred practice for varicocelectomy, laparoscopic or open procedures were excluded in this study. The short follow-up time was the fourth drawback (at least six months), which may underestimate the improvement rate of semen parameters. However, the typical duration to improve semen parameters following varicocelectomy is up to two spermatogenic cycles (less than six months) [21]. Fifth, we could not explore whether these predictors are associated with spontaneous pregnancy, which should be considered the ultimate goal of varicocelectomy; Therefore, further studies were needed to solve this issue. Finally, this study excluded patients with a history of previous varicocelectomy; thus, this nomogram cannot be applied to patients with recurrent varicocele. Despite the drawbacks, this is the first nomogram for predicting the improvement of the semen parameters in infertile men following MSV with excellent external validation. 

According to this study, the improvement of semen parameters in infertile men following MSV was significantly predicted by greater vein diameter and higher initial TPMSC. This nomogram aids in individualized decision making on the varicocele preoperative treatment plan and may help to enhance the therapeutic result.

## Figures and Tables

**Table 1:** Demographics and clinical data in this cohort according to the development cohort and validation cohort.

Variables	Development Cohort	Validation Cohort
Number of patients (%)	336 (100)	124 (100)
**Demographic characteristics**		
Age (years)	31.64 ± 4.28	31.44 ± 4.26
BMI (kg/m^2^)	25.32 ± 3.78	24.92 ± 3.80
Infertility duration (months)	24 (12, 36)	
**Dietary habits**		
Smoke (never vs. current or former)	246 (73.2)/90 (26.8)	93 (75.0)/31 (25.0)
**Varicocele**		
Surgical side (left vs. bilateral)	186 (55.4)/150 (44.6)	75 (60.5)/49 (39.5)
Grade of palpation (left, II vs. III)	156 (46.4)/180 (53.6)	55 (44.4)/69 (60.5)
Diameter of veins (left, mm)	3.33 ± 0.56	3.29 ± 0.56
Testicualr volume (left, mL)	15.34 ± 3.50	15.06 ± 3.54
**Semen parameters before MSV**		
Baseline TPMSC (10^6^)	13.82 ± 3.83	13.84 ± 3.72
Sperm concentration (10^6^/mL)	15.89 ± 2.87	15.31 ± 2.75
Morphology (normal forms, %)	2.84 ± 2.63	3.17 + 2.62
**Intraoperative data**		
Surgical approach (subinguinal vs. inguinal)	234 (69.6)/102 (30.4)	84 (67.7)/40 (32.3)
Ligated veins (number)	13.51 ± 4.36	13.08 ± 4.44

Continuous variables were expressed as median (interquartile range) or mean ± standard deviation; categorical variables were reported as number (percentage). Abbreviations: BMI, body mass index; MSV, microscopic varicocelectomy; TPMSC, total progressively motile sperm count. TPMSC = total sperm number (10^6^/ejaculate) × progressive motility (PR, %); PR, progressive (a + b motility).

**Table 2:** Demographics and clinical data in this cohort according to the semen parameters improvement type after MSV.

	Development Cohort 336 (100.00)			Validation Cohort 124 (100.00)		
Variables	No Improvement	Improvement	*p* Value	No Improvement	Improvement	*p* Value
Number of patients (%)	100 (29.8)	236 (70.2)		40 (32.3)	84 (67.7)	
**Demographic characteristics**						
Age (years)	31.72 ± 3.71	31.61 ± 4.51	0.830	31.33 ± 3.72	31.49 ± 4.38	0.839
BMI (kg/m^2^)	25.09 ± 4.11	25.07 ± 3.62	0.064	24.51 ± 4.13	25.11 ± 3.64	0.412
Infertility duration (months)	24 (24, 36)	24 (12, 36)	0.832	24 (24, 36)	24 (12, 36)	0.225
**Dietary habits**						
Smoke (never vs. current or former)	70 (70.0)/30 (30.0)	176 (74.6)/60 (25.4)	0.386	26 (65.0)/14 (35.0)	67 (79.8)/17 (20.2)	0.076
**Varicocele**						
Surgical side (left vs. bilateral)	54 (54.0)/46 (46.0)	132 (55.9)/104 (44.1)	0.745	21 (52.5)/19 (47.5)	54 (64.3)/30 (35.7)	0.210
Grade of palpation (left, II vs. III)	44 (44.0)/56 (56.0)	112 (47.5)/124 (52.5)	0.561	18 (45.0)/22 (55.0)	37 (44.0)/47 (56.0)	0.921
Diameter of veins (left, mm)	3.15 ± 0.49	3.41 ± 0.57	<0.001	3.13 ± 0.38	3.36 ± 0.62	0.033
Testicualr volume (left, mL)	14.82 ± 3.33	15.56 ± 3.55	0.076	14.00 ± 2.84	15.57 ± 3.74	0.020
**Semen parameters before MSV**						
Baseline TPMSC (10^6^)	9.82 ± 2.33	15.51 ± 2.98	<0.001	10.25 ± 2.25	15.55 ± 2.99	<0.001
Sperm concentration (10^6^/mL)	15.44 ± 2.79	16.08 ± 2.89	0.062	14.83 ± 2.32	15.55 ± 2.92	0.140
Morphology (normal forms, %)	2.60 ± 2.51	2.94 ± 2.68	0.279	3.02 ± 2.77	2.24 ± 2.56	0.674
**Intraoperative data**						
Surgical approach (subinguinal vs. inguinal)	72 (72.0)/28 (28.0)	162 (68.6)/74 (31.4)	0.541	28 (70.0)/12 (30.0)	56 (66.7)/28 (33.3)	0.711
Ligated veins (number)	13.34 ± 4.38	13.58 ± 4.49	0.650	13.10 ± 4.13	13.07 ± 4.60	0.973

Continuous variables were expressed as median (interquartile range) or mean ± standard deviation; categorical variables were reported as number (percentage). Independent samples Student’s *t*-test was used to compare mean of two continuous normally distributed variables and the Mann–Whitney U test was run to determine mean of two continuous non-normally distributed variables. Abbreviations: BMI, body mass index; MSV, microscopic varicocelectomy; TPMSC, total progressively motile sperm count. TPMSC = total sperm number (10^6^/ejaculate) × progressive motility (PR, %); PR, progressive (a + b motility).

**Table 3:** Multivariate binary logistic regression of semen parameter improvement after MSV.

Intercept and Variable	β	95% CI	OR	95% CI	*p*
Intercept	−13.598	−17.261, −9.934	1.24 × 10^−6^	3.19 × 10^−8^, 0.0000485	<0.001
Diameter of veins (left, mm)	1.181	0.382, 1.980	3.258	1.465, 7.242	0.004
Baseline TPMSC (10^6^)	0.885	0.682, 1.090	2.422	1.978, 2.966	<0.001
**Area under ROC curve**					
Development Dataset	0.942	0.918, 0.967	*p* < 0.001		
Validation Dataset	0.927	0.879, 0.974	*p* < 0.001		

The β coefficient, odds ratio, and 95% confidence interval were measured through binary logistic regression. Abbreviations: MSV, microscopic subinguinal varicocelectomy; OR, odds ratio; CI, confidence interval; TPMSC, total progressively motile sperm count. TPMSC = total sperm number (10^6^/ejaculate) × progressive motility (PR, %); PR, progressive (a + b motility); ROC, receiver operating characteristic curve.

## Data Availability

The data that support the findings of this study are available on request from the corresponding author. The data are not publicly available due to privacy or ethical restrictions.

## Supplementary Materials

The following supporting information can be downloaded at: https://www.mdpi.com/article/10.3390/jpm13010011/s1, Figure S1: Example of nomogram to predict semen parameters improvement after MSV.

## Abbreviations

MSV: microscopic varicocelectomy.
