# Supplementary material for: Nomogram for Predicting Semen Parameters Improvement after Microscopic Varicocelectomy in Infertile Men with Abnormal Semen Parameters
Source: J Pers Med. 2022 Dec 21;13(1):11. doi: 10.3390/jpm13010011 (PMC9865251; doi:10.3390/jpm13010011)
Supplement: Supplementary file 1 [file jpm-13-00011-s001.zip › jpm-2051021-supplementary.pdf]

Journal of
Personalized
Medicine

Supplementary Materials

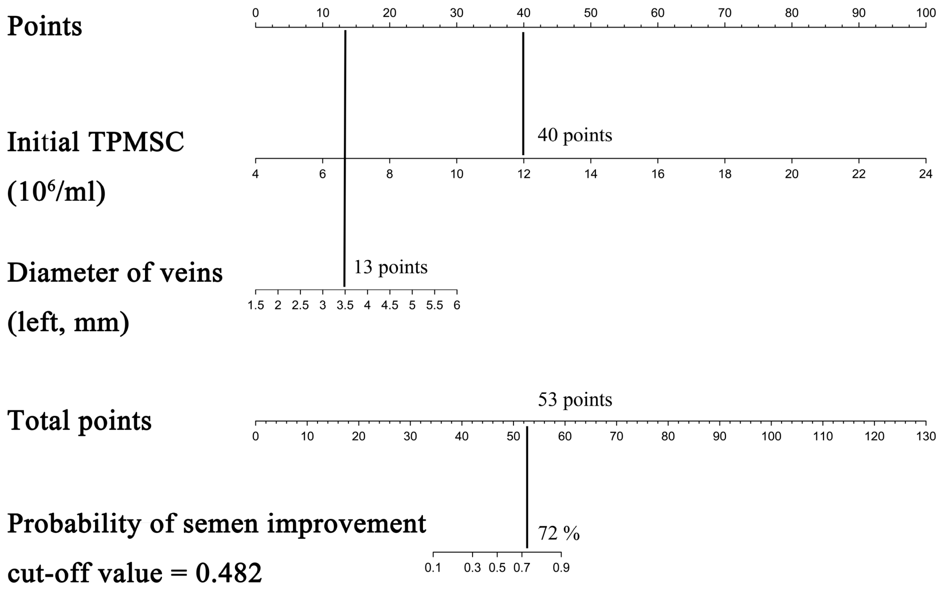

Figure S1. Example of nomogram to predict semen parameters improvement after MSV.
